# Mapping Sex-Specific Neurodevelopmental Alterations in Neurite Density and Morphology in a Rat Genetic Model of Psychiatric Illness

**DOI:** 10.1523/ENEURO.0426-20.2020

**Published:** 2021-03-09

**Authors:** Brian R. Barnett, Sue Y. Yi, McKenzie J. Poetzel, Keith Dodd, Nicholas A. Stowe, John-Paul J. Yu

**Affiliations:** 1 Neuroscience Training Program, Wisconsin Institutes for Medical Research, University of Wisconsin–Madison, Madison, WI 53705; 2 Department of Radiology, University of Wisconsin School of Medicine and Public Health, Madison, WI 53705; 3 Department of Biomedical Engineering, College of Engineering, University of Wisconsin–Madison, Madison, WI 53706; 4 Department of Psychiatry, University of Wisconsin School of Medicine and Public Health, Madison, WI 53705

**Keywords:** diffusion-weighted imaging, Disc1, DTI, MRI, NODDI, rat

## Abstract

Neurite orientation dispersion and density imaging (NODDI) is an emerging magnetic resonance (MR) diffusion-weighted imaging (DWI) technique that permits non-invasive quantitative assessment of neurite density and morphology. NODDI has improved our ability to image neuronal microstructure over conventional techniques such as diffusion tensor imaging (DTI) and is particularly suited for studies of the developing brain as it can measure and characterize the dynamic changes occurring in dendrite cytoarchitecture that are critical to early brain development. Neurodevelopmental alterations to the diffusion tensor have been reported in psychiatric illness, but it remains unknown whether advanced DWI techniques such as NODDI are able to sensitively and specifically detect neurodevelopmental changes in brain microstructure beyond those provided by DTI. We show, in an extension of our previous work with a *Disc1* svΔ2 rat genetic model of psychiatric illness, the enhanced sensitivity and specificity of NODDI to identify neurodevelopmental and sex-specific changes in brain microstructure that are otherwise difficult to observe with DTI and further corroborate observed changes in brain microstructure to differences in sex-specific systems-level animal behavior. Together, these findings inform the potential application and clinical translational utility of NODDI in studies of brain microstructure in psychiatric illness throughout neurodevelopment and further, the ability of advanced DWI methods such as NODDI to examine the role of biological sex and its influence on brain microstructure in psychiatric illness.

## Significance Statement

This research presents the first demonstration of the ability of neurite orientation dispersion and density imaging (NODDI) multicompartment diffusion imaging to uncover both neurodevelopmental and sex-specific alterations in brain microstructure in psychiatric illness. We show, in a genetic *Disc1* svΔ2 rat model, sex-specific neurodevelopmental patterns of neural microstructural change with NODDI and corresponding evidence of sex differences in behavioral endophenotypes of anxiety, cognition, and general activity. Together, our results support the potential impact and translational utility of NODDI to identify salient neurodevelopmental and sex-specific changes in brain microstructure in psychiatric illness beyond traditional morphometric and diffusion tensor approaches currently employed.

## Introduction

Animal models of psychiatric illness play a crucial role in furthering our understanding of the genetic, molecular, and microstructural (Gold et al., 2016; Gandal et al., 2018) features that contribute to the psychiatric disease state in numerous illnesses including autism spectrum disorder, schizophrenia, bipolar disorder, and major depressive disorder. As with other genetic variants that have been shown to confer an increased risk for psychiatric disease (Cross-Disorder Group of the Psychiatric Genomics Consortium et al., 2013), the balanced chromosomal t(1;11)(q42.1;q14.3) translocation of the *DISC1* gene has been implicated in several psychiatric illnesses including schizophrenia (Hodgkinson et al., 2004; Callicott et al., 2005; Hamshere et al., 2005), bipolar disorder (Hodgkinson et al., 2004), autism spectrum disorder (Kilpinen et al., 2008), and major depressive disorder (Hashimoto et al., 2006) and has emerged as a key biomolecular entry point toward understanding how shared genetic perturbations underpin a broad and diverse spectrum of psychiatric illness. Buttressing the longstanding interest and import of *DISC1* in neuroscience and neuropsychiatric research is a diverse repertoire of translational genetic animal models centered on the *DISC1* gene (Clapcote et al., 2007; Hikida et al., 2007). In addition to these murine models, a novel rat short genetic variant model of *DISC1* truncation (*Disc1* svΔ2) lacking exons 2–13 following targeted deletion with CRISPR/Cas9 has been generated and reported (Barnett et al., 2019). Importantly, owing to the larger brain volumes and thus greater signal-to-noise ratios that this model provides, this newly generated rat model is far more amenable to advanced magnetic resonance (MR) preclinical neuroimaging studies and crucially, can serve as a model neuroimaging system to test emerging advances in MR imaging (MRI) and to inform future clinical and translational imaging studies of neuropsychiatric illness.

While the majority of previously conducted investigations of neurodevelopmental alterations use measures of diffusion tensor imaging (DTI) and other diffusion-weighted imaging (DWI) techniques, it remains unknown whether newly developed advanced multicompartment DWI techniques such as neurite orientation dispersion and density imaging (NODDI) are able to sensitively and specifically detect neurodevelopmental changes in brain microstructure beyond those provided by conventional DWI techniques. NODDI represents an extension of single-compartment diffusion tensor models like DTI. Whereas quantitative indices of DTI such as fractional anisotropy (FA) are able to capture neural microstructural features but are inherently non-specific, multicompartment diffusion techniques can model water diffusion across multiple compartments that enable measurement of neurite density and orientation that represent biophysically relevant features in regions with variation in synaptic density and organization.

We sought to determine the ability of NODDI to detect both neurodevelopmental and sex-specific changes in brain microstructure in a *Disc1* svΔ2 rat genetic model and to corroborate changes in brain microstructure with systems-level behavioral studies. We report that male *Disc1* svΔ2 animals demonstrate a significant decrease in orientation dispersion [orientation dispersion index (ODI)] that are matched with concomitant deficits in measures of anxiety, hyperactivity, and cognition. We found evidence of strong sex-specific differences with female *Disc1* svΔ2 animals harboring significantly higher neurite density index (NDI) and ODI than sex-matched and age-matched controls while exhibiting no significant alterations to behavioral endophenotypes of anxiety or cognition. Taken together, our findings represent the first demonstration of NODDI diffusion imaging for the identification of sex-specific neurodevelopmental changes in brain microstructure in a genetic model of psychiatric illness and supports the clinical translational utility of NODDI for the study of brain microstructure beyond traditional morphometric and diffusion tensor approaches currently employed.

## Materials and Methods

### Subjects

Animals were housed and cared for in an Association for Assessment and Accreditation of Laboratory Animal Care (AAALAC)-accredited facility and all animal experiments were conducted in accordance with local Institutional Animal Care and Use Committee (IACUC)-approved protocols. Outbred control male and female Sprague Dawley rats (300–325 g, Charles River) and *Disc1* svΔ2 male and female rats (generated as described in Barnett et al., 2019) were pair housed in clear cages and were maintained under a 12/12 h light/dark cycle in humidity-controlled and temperature-controlled rooms with *ad libitum* access to food and water. Sprague Dawley pregnant dams were ordered from Charles River and all Sprague Dawley and *Disc1* svΔ2 male and female rats used in our data analyses were born, weaned, and matured to adulthood in the same housing facility. Animals were acclimated to housing conditions for 7 d before experimental manipulation. To generate the experimental *Disc1* svΔ2 animals, all *Disc1* svΔ2 male and female animals were generated from *Disc1* svΔ2 male-female homozygous pairings and subsequently genotyped to confirm genetic background (Barnett et al., 2019).

### Behavior analysis

Wild-type Sprague Dawley control rats and *Disc1* svΔ2 model rats (*n* = 43; 9 male and 10 female *Disc1* svΔ2 rats; 12 male and 12 female wild-type rats) at postnatal day (P)120–P150 were tested on the elevated-plus maze, Y-maze, and open field on three consecutive days. Sample sizes for behavioral assays were calculated according to prior power analyses from co-authors’ previous behavioral experiments. An hour before each day of testing, the animals were brought from their holding area to the experimental room to acclimate for 1 h. The same non-blinded experimenter handled the animals and conducted the behavioral assays to minimize experimenter variation. Blinding to the genotype of the animals undergoing testing was not necessary, as it did not affect parameter outcomes measured in these tasks. All animals underwent the same sequence of behavioral assays on each of the three consecutive days.

The open field task determines general activity levels, gross locomotor activity, and exploration habits. Assessment took place in a square, black plastic box. The animal was placed in the arena and allowed to freely move about for 10 min while being recorded by an overhead camera. The footage was then analyzed by an automated tracking system for the following parameters: distance moved, velocity, and time spent in predefined zones.

The Y-maze is a behavioral test for measuring the willingness of rodents to explore new environments. Rodents typically prefer to investigate a new arm of the maze rather than returning to one that was previously visited. Testing occurred in a Y-shaped maze with three black, opaque plastic arms at a 120° angle from each other. After introduction to the center of the maze, the animal could freely explore the three arms. Over the course of multiple arm entries, the subject should show a tendency to enter a less recently visited arm. The number of arm entries and the number of triads was recorded to calculate the percentage of alternation. An entry occurred when all four limbs are within the arm.

The elevated-plus maze is used to assess anxiety-related behavior. The elevated-plus maze apparatus consisted of a plus-shaped maze elevated above the floor with two oppositely positioned closed arms, two oppositely positioned open arms, and a center area. As subjects freely explored the maze, their behavior was recorded by means of a video camera mounted above the maze and analyzed using a video tracking system. The preference for being in open arms over closed arms (expressed as either as a percentage of entries and/or a percentage of time spent in the open arms) was calculated to measure anxiety-like behavior.

### Imaging methodology

Following the completion of the full battery of behavioral assays, outbred Sprague Dawley (control) rats and *Disc1* svΔ2 model rats (*n* = 24; six male and female per rat group) at an average age of P135 (full range P120–P150) were brought to a surgical plane of anesthesia before sequential transcardial perfusion with ice-cold PBS and 4% paraformaldehyde (PFA). The brains were cleanly dissected from the cranial vault and imaged with a 4.7 T Agilent MRI system and a 3.5-cm diameter quadrature volume RF coil. 3D multi-slice, diffusion weighted, spin echo protocols were used to acquire 10 non-diffusion weighted images (b = 0 s/mm^2^) and 75 diffusion-weighted images (25 non-colinear diffusion-weighting directions at b = 800 s/mm^2^, 50 non-colinear diffusion-weighting directions at b = 3500 s/mm^2^). Other imaging parameters: TE/TR = 24.17/2000 ms, FOV = 30 × 30 mm^2^, matrix = 192 × 192 reconstructed to 256 × 256 for an isotropic voxel size of 0.25 mm over two signal averages. Raw data files were converted to NIfTI (Neuroimaging Informatics Technology Initiative) format for use with the DTI-TK software package. Following correction for eddy currents and standard preprocessing (Smith et al., 2004), tensors were reconstructed, registered, and normalized to a study-specific template. NODDI modeling was performed with the microstructure diffusion toolbox (MDT; Harms et al., 2017) on a NVIDIA DGX-1 Deep Learning server (8-V100 GPUs, 32 GB RAM, Dual 20-core Intel Xeon E5-2698 v4 2.2 GHz CPUs and 512-GB system RAM) to remove run time constraints; analytical pipelines were specifically designed for imaging data collected from fixed *ex vivo* samples (e.g., using recommended diffusivity assumptions d∥ = 0.6 × 10^−3^ mm^2^/s and the d_iso_ = 2 × 10^−3^ mm^2^/s and using the “WatsonSHStickTortIsoVIsoDot_B0” fitting model as previously recommended; Zhang et al., 2012). Tract-based spatial statistics (TBSS) were then performed with permutation test results for multiple comparisons and threshold-free cluster enhancement (TFCE; Smith and Nichols, 2009) implemented with FSL’s Randomize where voxels were considered significant at the α < 0.05 level following family-wise error correction. Region of interest (ROI) analysis was conducted to examine specific areas selected a priori for their relevance to clinical psychiatric illness. TBSS and ROI analyses and statistical considerations are detailed in the Statistical analyses, below.

### Statistical analyses

For behavioral analyses, elevated-plus maze time in open arms and entries in open arms, Y-maze alternation percentage, and open field mean velocity, distance traveled, and time spent moving data were subjected to two-way ANOVA with genotype and sex as between-subjects factors. Tukey’s HSD *post hoc* comparison was used to detect differences at the *p *<* *0.05 level. The behavioral data analyzed meets the assumptions of normality and homogeneity of variances. Total sample size for ANOVA analysis was selected per prior recommended total sample sizes given an a priori effect size f = 0.5, α error probability = 0.05, and β = 0.8

For TBSS, a processing chain was adapted by replacing the standard TBSS registration (FSL’s FNIRT) with the DTI-TK registration routine. The TBSS pipeline was applied using the recommended parameters in FSL. An FA threshold of 0.2 was applied for the creation of the skeleton and a permutation test with *n* = 252, corrected for multiple comparisons and TFCE was implemented with FSL’s Randomize to compare each of the experimental groups to the control group, with *p *<* *0.05 as threshold for significance.

For ROI imaging analyses, the UNC P72 Rat Atlas was normalized to subject common space and masked with predefined ROIs. Diffusion measures for all ROIs from the atlas were extracted. Following automated volumetric segmentation of the brain, mean values of both diffusion and neurite indices were computed within six ROIs (hippocampus, external capsule, basal ganglia, internal capsule, neocortex, and corpus callosum) in each hemisphere for each individual subject. These ROIs were selected based on their relevance to mental illness for both major white matter and gray matter regions. Two-tailed, two-sample, and unequal variance Student’s *t* test was performed comparing FA, axial diffusion (AD), radial diffusion (RD), mean diffusivity (MD) [MD = (1/3)(TR); TR = trace diffusivity], NDI, and ODI mean values in *Disc1* svΔ2 animals against age-sex-matched controls. Raw *p* values were reported and adjusted *p* values to control for multiple comparisons were calculated using the Benjamini–Hochberg false discovery rate (FDR) correction (FDR = 0.05). Previous power analyses indicated observed effect size values of d = 2.5 or greater given low standard deviation between within-group replicates (σ between 0.01 and 0.001), validating sample sizes of six replicates per group. All data are available on reasonable request from the authors.

### Results

### *Disc1* svΔ2 harbors minimal voxel-wise changes in white matter microstructural integrity

**Figure 1.**
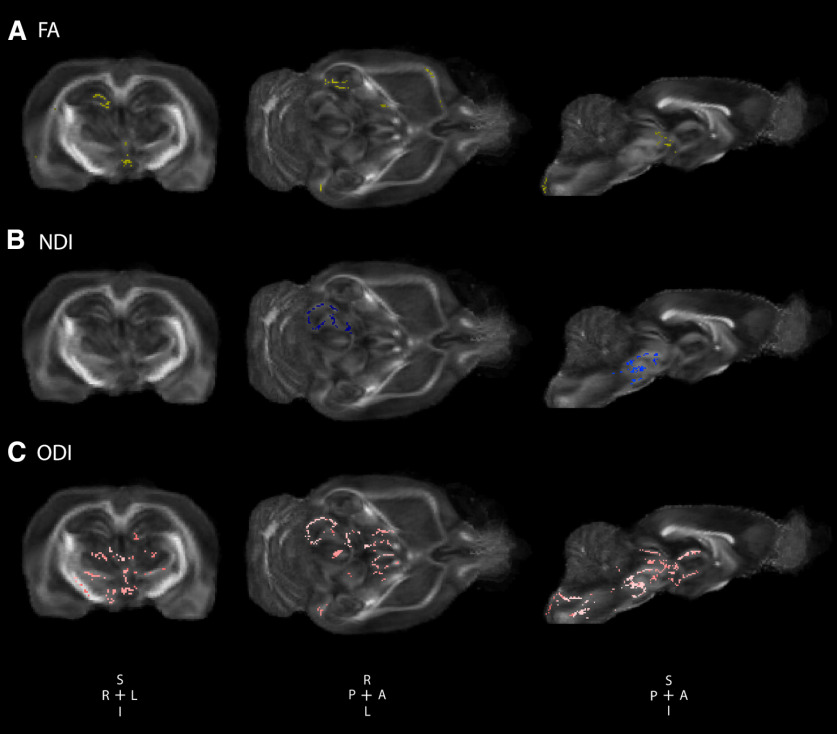
*Disc1* svΔ2 engenders significant sex-specific global alterations in orientation dispersion in males. *Disc1* svΔ2 in P120–P150 male rats demonstrate deficits in white matter microstructural integrity and contributes to global alterations in neurite density and orientation. ***A***, Voxel-wise tract-based spatial statistics significant areas of decreased FA in male *Disc1* svΔ2 rats (*n* = 6) compared with matched controls (*n* = 6; voxels in yellow). Representative coronal, axial, and sagittal sections reveal significant regions of decreased FA mainly in the right hippocampus, central hypothalamus, and right external capsule. ***B***, *Disc1* svΔ2 male rats demonstrated significant areas of decreased NDI compared with matched controls (voxels in blue). Representative coronal, axial, and sagittal sections reveal significant regions of decreased NDI predominantly in right substantia nigra. ***C***, *Disc1* svΔ2 male rats demonstrated significant areas of decreased ODI compared with matched controls (voxels in pink). Representative coronal, axial, and sagittal sections reveal significant regions of decreased ODI in the left hippocampus, bilateral thalamus, hypothalamus, and substantia nigra.

**Figure 2.**
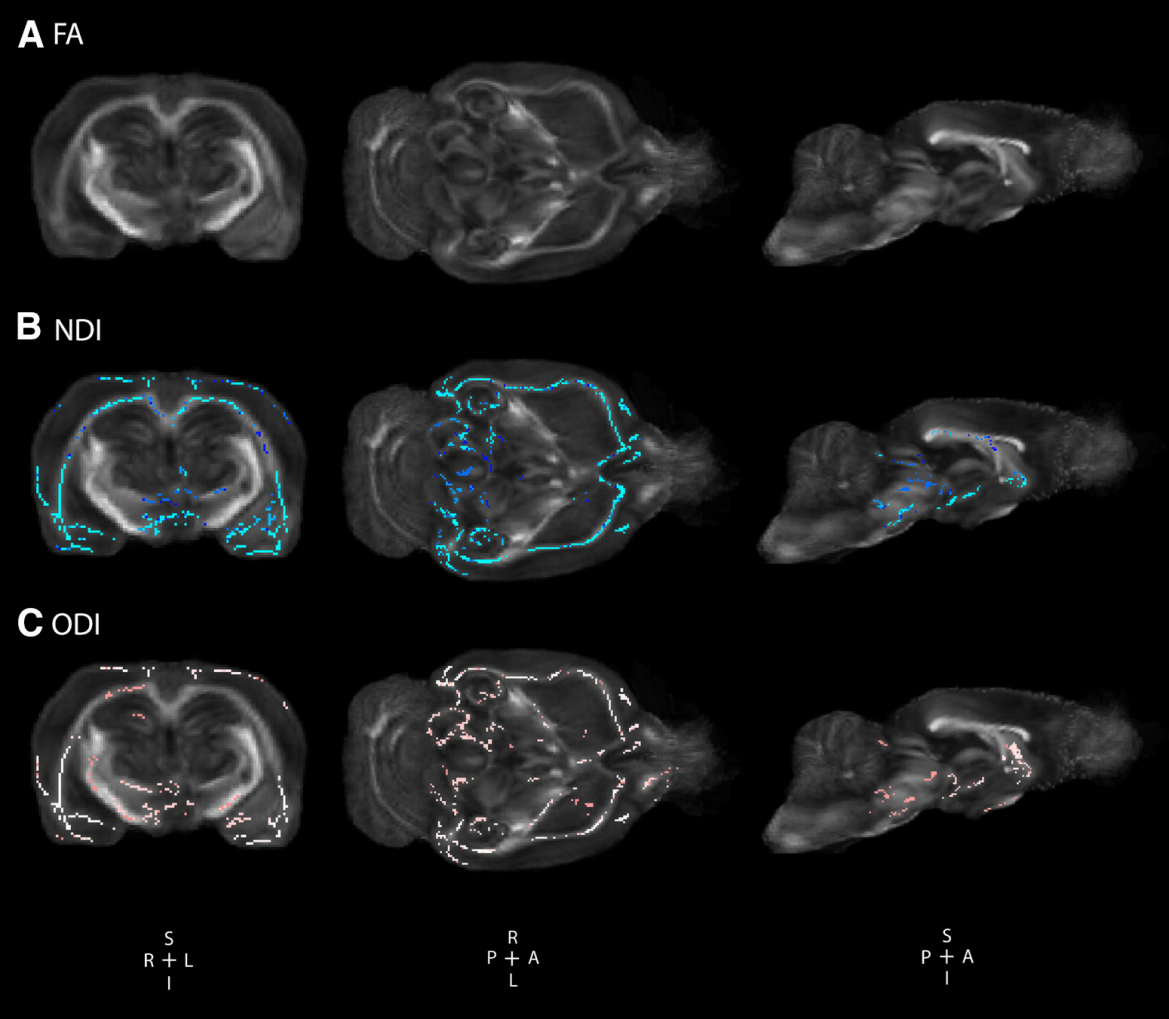
*Disc1* svΔ2 engenders significant sex-specific global alterations in neurite density and orientation in females. *Disc1* svΔ2 in P120–P150 female rats demonstrate significant increases in neurite density and orientation dispersion compared with matched controls. ***A***, Voxel-wise TBSS reveal no areas of significant difference in female *Disc1* svΔ2 rats (*n* = 6) compared with female controls (*n* = 6). ***B***, *Disc1* svΔ2 female rats demonstrated significant areas of increased NDI compared with female controls (voxels in blue). Representative coronal, axial, and sagittal sections show significant regions of increased NDI in neocortex, external capsule, corpus callosum, basal forebrain, thalamus, and hypothalamus. ***C***, *Disc1* svΔ2 female rats demonstrated significant increases in ODI compared with female controls (voxels in pink). Representative coronal, axial, and sagittal sections show significant regions of increased ODI in neocortex, external capsule, internal capsule, corpus callosum, basal forebrain, thalamus, and hypothalamus.

To explore and characterize the influence of early truncation of the major isoform of *Disc1* on white matter microstructure, *ex vivo* DTI was performed. Voxel-wise TBSS analysis of FA was performed comparing the *Disc1* svΔ2 model to age and sex-matched controls at a range of postnatal days from P120 to P150. TBSS analysis revealed that *Disc1* svΔ2 male rats harbor a minimal number of significant voxels of decreased FA when compared with matched controls (Fig. 1*A*). Similar to the male comparison, there were no significant voxel-wise differences in FA between *Disc1* svΔ2 females and matched wild-type controls (Fig. 2*A*).

### *Disc1* svΔ2 engenders significant sex-specific global alterations in neurite density and orientation

To further explore the role of *Disc1* on neural structure and organization, *ex vivo* NODDI was also performed. Voxel-wise TBSS analysis uncovered areas of decreased NDI and ODI values in male *Disc1* svΔ2 rats when compared with age and sex-matched controls (P120–P150). Decreased NDI values were seen in right substantia nigra and decreased ODI values were observed in left hippocampus and bilateral thalamus, hypothalamus, and substantia nigra (Fig. 1*B*,*C*). *Disc1* svΔ2 female rats demonstrated significant areas of increased NDI compared with female control rats in bilateral neocortex, external capsule, corpus callosum, basal forebrain, thalamus, and hypothalamus. *Disc1* svΔ2 female rats also demonstrated significant increases in ODI compared with female controls in neocortex, external capsule, internal capsule, corpus callosum, basal forebrain, thalamus, hypothalamus, and right hippocampus (Fig. 2*B*,*C*).

### *Disc1* svΔ2 contributes significant sex-specific changes in neural microstructure in regions salient to psychiatric illness

To a far greater degree than our DTI analysis, NODDI analyses sensitively capture microstructural differences in our *Disc1* svΔ2 model across the FA skeleton when compared with age and sex-matched controls. To further explore the impact of early truncation of *Disc1* in salient regions of the brain implicated in neuropsychiatric illnesses, a ROI analysis was performed. Six ROIs were a priori selected for further analysis: the neocortex, external capsule, corpus callosum, internal capsule, hippocampus, and basal ganglia (including the caudate, putamen, and globus pallidus). Following automated volumetric segmentation of the brain, mean values of both diffusion and neurite indices were computed within each ROI (left and right) for each individual subject for a total of 12 calculated ROIs per subject. *Disc1* svΔ2 male rats harbor significantly increased FA values in the bilateral external capsule, bilateral internal capsule, and right corpus callosum compared with age-matched and sex-matched controls. Additionally, *Disc1* svΔ2 male rats demonstrated significantly increased MD values in the right external capsule, right corpus callosum, and left internal capsule. *Disc1* svΔ2 male rats only demonstrated limited decreases in NDI, with a significant decrease in the right hippocampus compared with control male rats. Finally, *Disc1* svΔ2 male rats had significantly reduced ODI in the bilateral hippocampus, bilateral external capsule, and left internal capsule (Table 1). Stricter analyses controlling for multiple comparisons (FDR with the Benjamini–Hochberg procedure with an FDR set to 0.05) sustained the significant difference findings for FA in the left internal capsule and ODI in the right external capsule and left internal capsule. *Disc1* svΔ2 female rats demonstrated significantly decreased FA in the bilateral neocortex and left basal ganglia ROIs as well as significantly decreased MD in bilateral hippocampus, left external capsule, left basal ganglia, right internal capsule, and bilateral neocortex. In a reversal of the direction of changes in neural microstructure, significant increases in NDI were observed in bilateral hippocampus, external capsule, basal ganglia, neocortex, as well as right internal capsule. *Disc1* svΔ2 female rats also had significantly increased ODI in bilateral hippocampus, external capsule, and neocortex, as well as in left basal ganglia and right internal capsule ROIs (Table 1). Of these significant results, the findings for FA in the left basal ganglia and left neocortex, NDI in bilateral hippocampus, external capsule, neocortex, and left basal ganglia, and ODI in bilateral hippocampus, neocortex, right external capsule, and left basal ganglia were significant after applying the FDR procedure.

**Table 1:** *Disc1 svΔ2* contributes to sex-specific significant changes in neural microstructure in salient regions implicated in psychiatric illness

DTI measure	Hemi	ROI	Mean (±SEM)		*p* value	Mean (±SEM)		*p* value
			Control male	Disc1 svΔ2 male	Male	Control female	Disc1 svΔ2 female	Female
FA	Right	HC	0.22696 (±0.00393)	0.23499 (±0.00313)	0.071	0.22195 (±0.00161)	0.22138 (±0.00475)	0.456
		EC	0.43209 (±0.00495)	0.44551 (±0.00385)	<0.05^a^	0.41092 (±0.00825)	0.41625 (±0.00615)	0.308
		BG	0.17118 (±0.00348)	0.17224 (±0.00498)	0.433	0.16650 (±0.00405)	0.16134 (±0.00511)	0.224
		IC	0.35172 (±0.00915)	0.37733 (±0.00336)	<0.05^a^	0.34653 (±0.00520)	0.35081 (±0.00597)	0.300
		NC	0.20396 (±0.00323)	0.20222 (±0.00126)	0.314	0.19598 (±0.00312)	0.18757 (±0.00216)	<0.05^a^
		CC	0.57858 (±0.01005)	0.60223 (±0.00361)	<0.05^a^	0.55144 (±0.01868)	0.55118 (±0.01252)	0.496
	Left	HC	0.22532 (±0.00451)	0.23356 (±0.00281)	0.077	0.21396 (±0.00229)	0.21364 (±0.00605)	0.481
		EC	0.41523 (±0.00582)	0.42855 (±0.00279)	<0.05^a^	0.39655 (±0.00768)	0.40232 (±0.00569)	0.280
		BG	0.18215 (±0.00368)	0.187 (±0.00259)	0.154	0.17617 (±0.00279)	0.16496 (±0.00326)	<0.05^a^^,b^
		IC	0.59219 (±0.00823)	0.62837 (±0.00843)	<0.01^a^^,b^	0.62386 (±0.00768)	0.62077 (±0.00764)	0.391
		NC	0.20278 (±0.00302)	0.20187 (±0.00276)	0.414	0.19805 (±0.00229)	0.18935 (±0.00219)	<0.05^a^^,b^
		CC	0.58245 (±0.01006)	0.59938 (±0.00633)	0.093	0.54712 (±0.01647)	0.55733 (±0.01176)	0.312
MD	Right	HC	0.38200 (±0.00305)	0.39350 (±0.00594)	0.058	0.41216 (±0.00286)	0.39116 (±0.00724)	<0.05^a^^,b^
		EC	0.29550 (±0.00393)	0.30550 (±0.00354)	<0.05^a^	0.32133 (±0.00423)	0.30833 (±0.01012)	0.132
		BG	0.35850 (±0.00642)	0.37150 (±0.01014)	0.152	0.37466 (±0.00419)	0.36366 (±0.00762)	0.117
		IC	0.31833 (±0.00514)	0.33150 (±0.00744)	0.088	0.34983 (±0.0029)	0.32733 (±0.0116)	<0.05^a^
		NC	0.37100 (±0.00251)	0.37683 (±0.00582)	0.190	0.40100 (±0.00382)	0.36583 (±0.00438)	<0.01^a^^,b^
		CC	0.23950 (±0.00255)	0.25233 (±0.00352)	<0.01^a^^,b^	0.26116 (±0.00423)	0.26500 (±0.01066)	0.373
	Left	HC	0.38316 (±0.00396)	0.39716 (±0.00683)	0.053	0.41300 (±0.00263)	0.38983 (±0.00517)	<0.01^a^^,b^
		EC	0.29833 (±0.00358)	0.30800 (±0.00508)	0.076	0.33433 (±0.00276)	0.31033 (±0.00486)	<0.001^a^^,b^
		BG	0.36000 (±0.00543)	0.37216 (±0.01100)	0.172	0.38200 (±0.00372)	0.36433 (±0.00624)	<0.05^a^
		IC	0.25366 (±0.00140)	0.27850 (±0.00407)	<0.01^a^^,b^	0.27733 (±0.00322)	0.27216 (±0.00855)	0.292
		NC	0.37333 (±0.0016)	0.38183 (±0.00611)	0.104	0.40150 (±0.00350)	0.36900 (±0.00405)	<0.001^a^^,b^
		CC	0.23933 (±0.00374)	0.23950 (±0.00313)	0.487	0.25433 (±0.00422)	0.25333 (±0.0093)	0.462
NDI	Right	HC	0.28863 (±0.00618)	0.26707 (±0.00871)	<0.05^a^	0.24767 (±0.00455)	0.28407 (±0.00956)	<0.01^a^^,b^
		EC	0.45984 (±0.00571)	0.45174 (±0.00608)	0.177	0.42192 (±0.00873)	0.46351 (±0.00943)	<0.01^a^^,b^
		BG	0.31123 (±0.01085)	0.29408 (±0.01434)	0.181	0.29221 (±0.00516)	0.31111 (±0.00865)	<0.05^a^
		IC	0.40446 (±0.01286)	0.4146 (±0.01761)	0.326	0.38355 (±0.00917)	0.41161 (±0.01145)	<0.05^a^
		NC	0.30812 (±0.00400)	0.30277 (±0.00925)	0.303	0.26590 (±0.00731)	0.32010 (±0.00613)	<0.001^a^^,b^
		CC	0.55335 (±0.00622)	0.54253 (±0.00564)	0.114	0.51977 (±0.00761)	0.53613 (±0.00934)	0.102
	Left	HC	0.28052 (±0.00733)	0.26581 (±0.00928)	0.088	0.24657 (±0.00283)	0.28127 (±0.00719)	<0.001^a^^,b^
		EC	0.46140 (±0.00636)	0.45002 (±0.00541)	0.102	0.40485 (±0.00641)	0.44984 (±0.00804)	<0.001^a^^,b^
		BG	0.31546 (±0.00837)	0.29646 (±0.01709)	0.171	0.28374 (±0.00405)	0.30895 (±0.00511)	<0.001^a^^,b^
		IC	0.56072 (±0.00828)	0.57002 (±0.01176)	0.266	0.59342 (±0.00829)	0.57928 (±0.01304)	0.191
		NC	0.31333 (±0.00497)	0.30039 (±0.00861)	0.111	0.27232 (±0.00650)	0.31719 (±0.00547)	<0.001^a^^,b^
		CC	0.56460 (±0.00551)	0.55457 (±0.00409)	0.087	0.52610 (±0.00581)	0.55257 (±0.01351)	0.051
ODI	Right	HC	0.22756 (±0.00696)	0.19927 (±0.00745)	<0.01^a^	0.18556 (±0.00531)	0.22146 (±0.00522)	<0.001^a^^,b^
		EC	0.16645 (±0.00415)	0.15200 (±0.00203)	<0.01^a^^,b^	0.14986 (±0.00438)	0.16932 (±0.00527)	<0.01^a^^,b^
		BG	0.32012 (±0.01118)	0.30247 (±0.01745)	0.207	0.30554 (±0.01219)	0.33371 (±0.01499)	0.088
		IC	0.20721 (±0.01170)	0.18788 (±0.00966)	0.116	0.17752 (±0.00949)	0.20551 (±0.00673)	<0.05^a^
		NC	0.27603 (±0.00609)	0.27409 (±0.00876)	0.430	0.23920 (±0.01224)	0.30206 (±0.0077)	<0.01^a^^,b^
		CC	0.11298 (±0.00792)	0.10064 (±0.00210)	0.082	0.11605 (±0.01085)	0.11905 (±0.01174)	0.427
	Left	HC	0.22566 (±0.01036)	0.19509 (±0.00768)	<0.05^a^	0.19621 (±0.00513)	0.23155 (±0.00736)	<0.01^a^^,b^
		EC	0.17835 (±0.00486)	0.16079 (±0.00440)	<0.05^a^	0.15204 (±0.00750)	0.17760 (±0.00740)	<0.05^a^
		BG	0.30551 (±0.00904)	0.28399 (±0.01504)	0.124	0.28359 (±0.00900)	0.32845 (±0.01229)	<0.01^a^^,b^
		IC	0.10624 (±0.00241)	0.08015 (±0.00521)	<0.001^a^^,b^	0.07701 (±0.00452)	0.08432 (±0.00457)	0.141
		NC	0.28466 (±0.00530)	0.27898 (±0.01100)	0.326	0.24563 (±0.01062)	0.30203 (±0.00728)	<0.001^a^^,b^
		CC	0.12171 (±0.00843)	0.11224 (±0.00687)	0.202	0.12680 (±0.01136)	0.12966 (±0.00821)	0.421

All values are mean ± SEM. Units of measure for FA, MD, NDI, and ODI are 10^3^ mm^2^/s. ROIs correspond to ROIs derived from the P72 UNC Atlas. Diffusion measure abbreviations: Hemi = hemisphere; FA = fractional anisotropy; MD = mean diffusivity, NDI = neurite density index; ODI = orientation dispersion index. ROI abbreviations: HC = hippocampus; EC = external capsule; BG = basal ganglia; IC = internal capsule; NC = neocortex; CC = corpus callosum. For all sample groups, *n *=* *6.

a Statistically significant.

b Statistically significant after controlling the FDR with the Benjamini–Hochberg procedure (FDR = 0.05).

In addition to the statistical analyses comparing *Disc1* svΔ2 and control ROIs within male and female rats, additional statistical analyses directly comparing *Disc1* svΔ2 male rats to *Disc1* svΔ2 female rats found significantly higher FA in male rats and significantly higher ODI in female rats. *Disc1* svΔ2 male rats demonstrated significantly higher FA in bilateral hippocampus, external capsule, neocortex, corpus callosum, left basal ganglia, and right internal capsule. *Disc1* svΔ2 female rats demonstrated significantly higher ODI in bilateral hippocampus, external capsule, right neocortex, and left basal ganglia. There were no significant differences between male *Disc1* svΔ2 and female *Disc1* svΔ2 rats for MD or NDI (Table 2).

**Table 2:** Intersex comparison of *Disc1 svΔ2* neural microstructure

DTI measure	Hemi	ROI	Mean (±SEM)		*p* value
			Disc1 svΔ2 female	Disc1 svΔ2 male	
FA	Right	HC	0.22138 (±0.00475)	0.23499 (±0.00313)	<0.05^a^^,b^
		EC	0.41625 (±0.00615)	0.44551 (±0.00385)	<0.01^a^^,b^
		BG	0.16134 (±0.00511)	0.17224 (±0.00498)	0.079
		IC	0.35081 (±0.00597)	0.37733 (±0.00336)	<0.01^a^^,b^
		NC	0.18757 (±0.00216)	0.20222 (±0.00126)	<0.01^a^^,b^
		CC	0.55118 (±0.01252)	0.60223 (±0.00361)	<0.01^a^^,b^
	Left	HC	0.21364 (±0.00605)	0.23356 (±0.00281)	<0.01^a^^,b^
		EC	0.40232 (±0.00569)	0.42855 (±0.00279)	<0.01^a^^,b^
		BG	0.16496 (±0.00326)	0.18700 (±0.00259)	<0.01^a^^,b^
		IC	0.62077 (±0.00764)	0.62837 (±0.00843)	0.260
		NC	0.18935 (±0.00219)	0.20187 (±0.00276)	<0.01^a^^,b^
		CC	0.55733 (±0.01176)	0.59938 (±0.00633)	<0.01^a^^,b^
MD	Right	HC	0.39116 (±0.00724)	0.39350 (±0.00594)	0.404
		EC	0.30833 (±0.01012)	0.30550 (±0.00354)	0.399
		BG	0.36366 (±0.00762)	0.37150 (±0.01014)	0.275
		IC	0.32733 (±0.0116)	0.33150 (±0.00744)	0.384
		NC	0.36583 (±0.00438)	0.37683 (±0.00582)	0.081
		CC	0.26500 (±0.01066)	0.25233 (±0.00352)	0.143
	Left	HC	0.38983 (±0.00517)	0.39716 (±0.00683)	0.206
		EC	0.31033 (±0.00486)	0.30800 (±0.00508)	0.374
		BG	0.36433 (±0.00624)	0.37216 (±0.01100)	0.275
		IC	0.27216 (±0.00855)	0.27850 (±0.00407)	0.259
		NC	0.36900 (±0.00405)	0.38183 (±0.00611)	0.055
		CC	0.25333 (±0.0093)	0.23950 (±0.00313)	0.095
NDI	Right	HC	0.28407 (±0.00956)	0.26707 (±0.00871)	0.109
		EC	0.46351 (±0.00943)	0.45174 (±0.00608)	0.160
		BG	0.31111 (±0.00865)	0.29408 (±0.01434)	0.167
		IC	0.41161 (±0.01145)	0.4146 (±0.01761)	0.445
		NC	0.32010 (±0.00613)	0.30277 (±0.00925)	0.075
		CC	0.53613 (±0.00934)	0.54253 (±0.00564)	0.285
	Left	HC	0.28127 (±0.00719)	0.26581 (±0.00928)	0.109
		EC	0.44984 (±0.00804)	0.45002 (±0.00541)	0.493
		BG	0.30895 (±0.00511)	0.29646 (±0.01709)	0.250
		IC	0.57928 (±0.01304)	0.57002 (±0.01176)	0.305
		NC	0.31719 (±0.00547)	0.30039 (±0.00861)	0.065
		CC	0.55257 (±0.01351)	0.55457 (±0.00409)	0.445
ODI	Right	HC	0.22146 (±0.00522)	0.19927 (±0.00745)	<0.05^a^^,b^
		EC	0.16932 (±0.00527)	0.15200 (±0.00203)	<0.01^a^^,b^
		BG	0.33371 (±0.01499)	0.30247 (±0.01745)	0.102
		IC	0.20551 (±0.00673)	0.18788 (±0.00966)	0.083
		NC	0.30206 (±0.0077)	0.27409 (±0.00876)	<0.05^a^^,b^
		CC	0.11905 (±0.01174)	0.10064 (±0.00210)	0.077
	Left	HC	0.23155 (±0.00736)	0.19509 (±0.00768)	<0.01^a^^,b^
		EC	0.17760 (±0.00740)	0.16079 (±0.00440)	<0.05^a^
		BG	0.32845 (±0.01229)	0.28399 (±0.01504)	<0.05^a^
		IC	0.08432 (±0.00457)	0.08015 (±0.00521)	0.280
		NC	0.30203 (±0.00728)	0.27898 (±0.01100)	0.056
		CC	0.12966 (±0.00821)	0.11224 (±0.00687)	0.067

All values are mean ± SEM. Units of measure for FA, MD, NDI, and ODI are 10^3^ mm^2^/s. ROIs correspond to ROIs derived from the P72 UNC Atlas. Diffusion measure abbreviations: Hemi = hemisphere; FA = fractional anisotropy; MD = mean diffusivity, NDI = neurite density index; ODI = orientation dispersion index. ROI abbreviations: HC = hippocampus; EC = external capsule; BG = basal ganglia; IC = internal capsule; NC = neocortex; CC = corpus callosum. For all sample groups, *n *=* *6.

a Statistically significant.

b Statistically significant after controlling the FDR with the Benjamini–Hochberg procedure (FDR = 0.05).

### *Disc1* svΔ2 behavioral endophenotypes reinforce patterns of sex-specific alteration in neural microstructure

**Figure 3.**
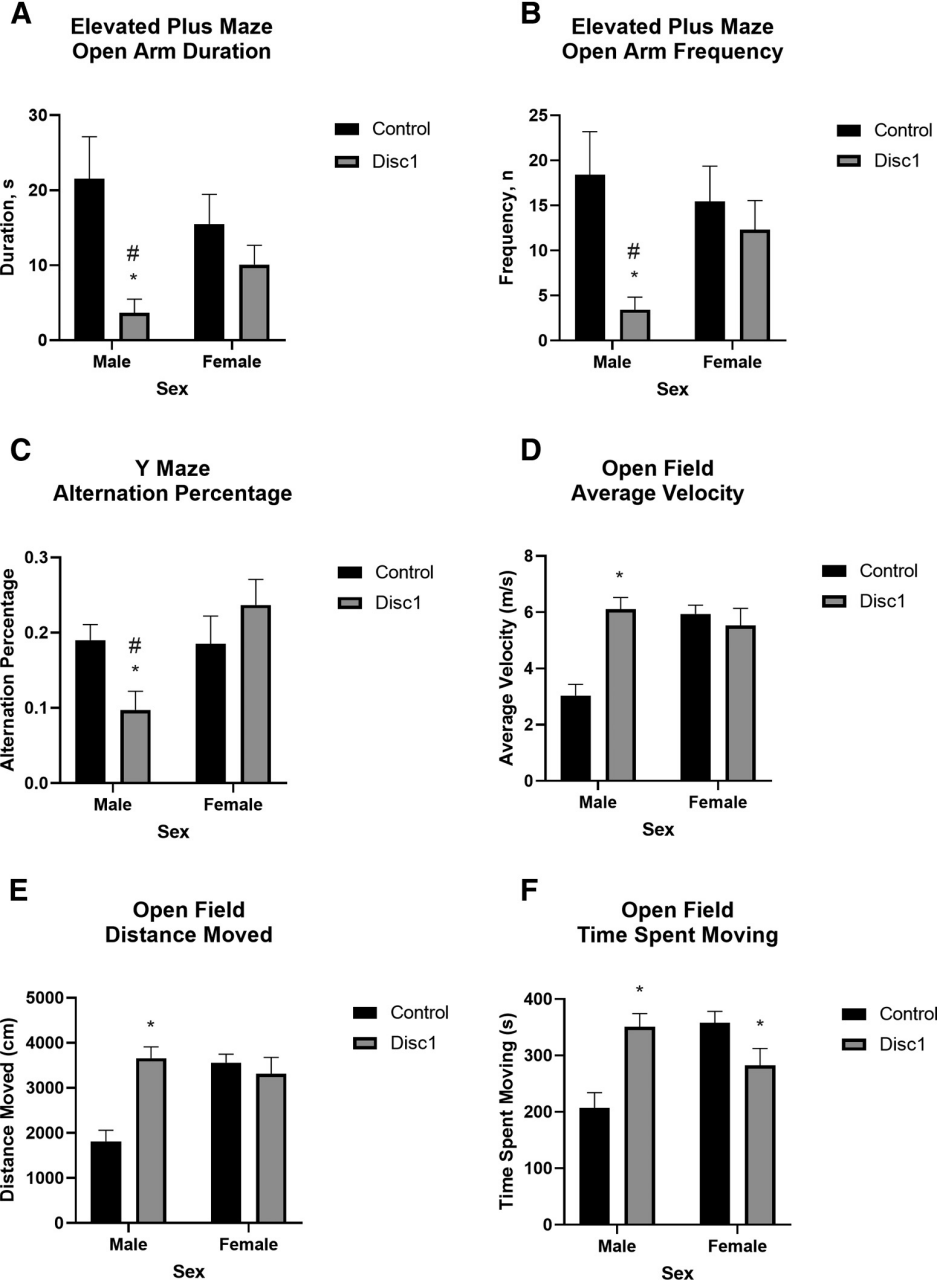
*Disc1* svΔ2 behavioral endophenotypes reinforce patterns of sex-specific alteration in neural microstructure. ***A***, Mean duration in open arms of the elevated-plus maze (±SEM) of wild-type male (*n* = 12), *Disc1* svΔ2 male (*n* = 9), wild-type female (*n* = 12), and *Disc1* svΔ2 female (*n* = 10) rats. There was a significant effect of genotype (*F*_(1,39)_ = 8.081, *p *= 0.007). Mean duration was significantly shorter in *Disc1* svΔ2 males versus wild-type males (*p *= 0.007), denoted by *. Mean duration was significantly shorter in *Disc1* svΔ2 males versus *Disc1* svΔ2 females (*p *= 0.032), denoted by #. ***B***, Mean frequency in open arms of the elevated-plus maze (±SEM) of wild-type male (*n* = 12), *Disc1* svΔ2 male (*n* = 9), wild-type female (*n* = 12), and *Disc1* svΔ2 female (*n* = 10) rats. There was a significant effect of genotype (*F*_(1,39)_ = 5.476, *p *= 0.025). Mean frequency was significantly lower in *Disc1* svΔ2 males versus wild-type males (*p *= 0.008), denoted by *. Mean frequency was significantly lower in *Disc1* svΔ2 males versus *Disc1* svΔ2 females (*p *= 0.013), denoted by #. ***C***, Mean alternation percentage in the y-maze (±SEM) of wild-type male (*n* = 12), *Disc1* svΔ2 male (*n* = 9), wild-type female (*n* = 12), and *Disc1* svΔ2 female (*n* = 10) rats. There was a significant effect of sex (*F*_(1,39)_ = 4.875, *p *= 0.033) and genotype × sex interaction (*F*_(1,39)_ = 5.620, *p *= 0.023). Mean alternation percentage was significantly lower in *Disc1* svΔ2 males versus wild-type males (*p *= 0.005), denoted by *. Mean alternation percentage was significantly lower in *Disc1* svΔ2 males versus *Disc1* svΔ2 females (*p *= 0.002), denoted by #. ***D***, Mean open field velocity (±SEM) of wild-type male (*n* = 12), *Disc1* svΔ2 male (*n* = 9), wild-type female (*n* = 12), and *Disc1* svΔ2 female (*n* = 10) rats. There was a significant effect of genotype (*F*_(1,39)_ = 9.125, *p *= 0.004), sex (*F*_(1,39)_ = 7.003, *p *= 0.012), and genotype × sex interaction (*F*_(1,39)_ = 15.65, *p *< 0.001). Mean velocity was significantly higher in *Disc1* svΔ2 males versus wild-type males (*p *< 0.001), denoted by *. ***E***, Mean open field distance traveled (±SEM) of wild-type male (*n* = 12), *Disc1* svΔ2 male (*n* = 9), wild-type female (*n* = 12), and *Disc1* svΔ2 female (*n* = 10) rats. There was a significant effect of genotype (*F*_(1,39)_ = 9.134, *p *= 0.004), sex (*F*_(1,39)_ = 7.000, *p *= 0.012), and genotype × sex interaction (*F*_(1,39)_ = 15.71, *p *< 0.001). Mean distance traveled was significantly higher in *Disc1* svΔ2 males versus wild-type males (*p *< 0.001), denoted by *. ***F***, Mean open field time spent moving (±SEM) of wild-type male (*n* = 12), *Disc1* svΔ2 male (*n* = 9), wild-type female (*n* = 12), and *Disc1* svΔ2 female (*n* = 10) rats. There was a significant genotype × sex interaction (*F*_(1,39)_ = 18.54, *p *< 0.001). Mean distance traveled was significantly higher in *Disc1* svΔ2 males versus wild-type males (*p *< 0.001) and in *Disc1* svΔ2 females versus wild-type females (*p *= 0.022), denoted by *.

*Disc1* svΔ2 male rats exhibited a consistent pattern of behavioral impairments at the P120–P150 time point. We investigated whether *Disc1* svΔ2 rats exhibited anxious-like behavior in the elevated plus maze, which has two opposed closed arms with high walls and two opposed open arms without walls. The duration of time on and frequency of entry into the open arms was significantly lower in *Disc1* svΔ2 male rats than in wild-type male rats, as well as in *Disc1* svΔ2 male rats versus *Disc1* svΔ2 female rats (Fig. 3*A*,*B*). We assessed working memory using a free choice three-arm Y-maze. Alternations, consisting of entry into each of the three arms in succession, were recorded as a percentage of the maximum number of alternations possible (alternation percentage). The alternation percentage was significantly lower in *Disc1* svΔ2 male rats than in wild-type male rats, as well as in *Disc1* svΔ2 male rats versus *Disc1* svΔ2 female rats (Fig. 3*C*). In a novel, open field, *Disc1* svΔ2 male rats exhibited significantly higher locomotion, composed of average velocity, distance traveled, and time spent moving, than wild-type male rats, while *Disc1* svΔ2 female rats exhibited significantly lower time spent moving than wild-type female rats (Fig. 3*D–F*). That *Disc1* svΔ2 female rats display significantly lower time spent moving, concomitantly with a statistically similar average velocity and distance traveled compared with wild-type female rats, suggests that these *Disc1* svΔ2 female rats displayed greater locomotion speed and distance covered throughout the 10-min testing period when they were actually moving.

## Discussion

Here, we demonstrate the ability of NODDI to highlight neurodevelopmental trajectories and differentiate sex-specific changes in brain microstructure that are otherwise difficult to observe with DTI and further corroborate these changes with observed sex-specific differences in systems-level animal behavior. These findings inform the potential application and clinical translational utility of NODDI in studies of brain microstructure in psychiatric illness throughout neurodevelopment and further, the ability of advanced DWI methods such as NODDI to examine the role of biological sex and its influence on brain microstructure in psychiatric illness. Longitudinal or cross-sectional experimental designs incorporating multiple neurodevelopmental timepoints are essential to capture the degree and spatial distribution of neural microstructural change that occurs over different stages of mental illness (Bracht et al., 2015; Herringa, 2017; Seitz et al., 2018). Despite this, longitudinal imaging studies of psychiatric illness across the neurodevelopmental spectrum are relatively rare; however, the few available cross-sectional analyses at different age points have suggested that some DWI methods can identify age-dependent pathologic changes over the course of disease development (Pasternak et al., 2012, 2015). While these studies largely describe neurodevelopmental alterations in measures of DTI and other DWI techniques, the findings presented herein provide insight into the potential for NODDI to sensitively and specifically detect neurodevelopmental changes in brain microstructure beyond those provided by conventional DWI techniques.

The relative advantages provided by NODDI compared with traditional FA analyses is exemplified by the *Disc1* svΔ2 male neuroimaging findings. Surprisingly, *Disc1* svΔ2 male rats at P120–P150 demonstrate a minimal number of significant voxels of decreased FA when compared with matched controls with TBSS analysis. This finding comes in contrast to previously observed global FA decreases in *Disc1* svΔ2 male rats when compared with matched controls at a P84 time point with TBSS analysis (Barnett et al., 2019). Additionally, on a ROI basis, *Disc1* svΔ2 male rats did have significantly increased FA values in the bilateral external capsule, bilateral internal capsule, and left corpus callosum compared with age-matched and sex-matched controls. These ROI findings indicate greater potential specificity to FA alteration than can be observed with TBSS voxel-wise analyses, which can partly be attributed to the TBSS technique itself, which considers maximum differences in FA in neighboring voxels in the FA skeleton as opposed to an ROI approach, which accounts for differences in average FA in all voxels in the ROI. The *Disc1* svΔ2 male ROI findings indicate surprising differences in FA from P84 to P120–P150 specifically in major white matter fiber tracts. Prior DTI studies describe FA decreases in frontal commissural and association fiber tracts in human DISC1 t(1;11) translocation carriers and in DISC1 Ser704Cys SNP allele carriers at adult time points (Sprooten et al., 2011; Whalley et al., 2015). Our findings of increased FA at this time point stand as part of a complex axis of findings in regards to changes to FA where prior work in our laboratory and many studies of the human clinical population indicate decreased FA, while other findings present increased FA in schizophrenia patients especially in subcortical white matter (Seok et al., 2007; Rotarska-Jagiela et al., 2009; Alba-Ferrara and de Erausquin, 2013). These findings demonstrate the potential value of assaying multiple timepoints to identify dynamic neurodevelopmental transformations as well as the collection of parameters other than FA to assess other changes in neural microstructure that may also be occurring. The findings of increased FA and MD in white matter tracts in these ROI analyses in *Disc1* svΔ2 males may represent a pattern of deficient axonal pruning that has been previously reported in autism spectrum disorder (Valnegri et al., 2017; Suetterlin et al., 2018; Oldehinkel et al., 2019; Pulikkan et al., 2019), while observed decreases to ODI in combination may indicate that these deficiencies may be redundant and inefficient without a normal orientation dispersion of axons. As DISC1 regulates development of synaptic growth and trans-synaptic structure, organization, and function, it would be predicted to impact neuroimaging measures of neurite density and orientation (Camargo et al., 2007; Brandon and Sawa, 2011; Hikida et al., 2012; Furukubo-Tokunaga et al., 2016; Unda et al., 2016). As anticipated, male *Disc1 svΔ2* rats harbor decreased orientation dispersion across the selected ROIs, consistent with previous studies of both dysmorphic and decreased dendritic density and arborization as seen in models of both *Disc1* underexpression and overexpression (Miyoshi et al., 2003; Ozeki et al., 2003).

For female animals, at the P120–P150 time point, there were also no significant TBSS differences in FA values between *Disc1* svΔ2 females and matched wild-type controls. In contrast, ROI analyses revealed that *Disc1* svΔ2 female rats demonstrated significantly decreased FA and MD in multiple ROIs. The *Disc1* svΔ2 female rats only demonstrate FA decreases in predominantly gray matter regions at the current time point compared with a more global TBSS and ROI-based observation of decreased FA at the P84 time point. These ROI findings reiterate the finding of greater specificity to FA decreases that can be observed with ROI analyses compared with TBSS voxel-wise analyses. Unexpected sex-specific differences were also evident in the distribution of voxel-wise change in measures of NDI and ODI. *Disc1* svΔ2 female rats demonstrated significantly increased NDI and ODI values (Fig. 2*B*,*C*). Previous analyses at P84 in *Disc1* svΔ2 female rats indicated that NDI was reduced while ODI was not changed at all in comparison to matched controls, and our current results indicate an observed increase in NDI and ODI measures. Previous study of measures of neurite density and orientation dispersion in the context of schizophrenia and first episode psychosis have observed significant decreases in NDI across a range of interhemispheric, corticospinal, and association tracts, but the studies did not analyze sex differences between male and female participants (Nazeri et al., 2017; Rae et al., 2017). These findings of greater and more diffuse increases to NDI and ODI matter microstructural integrity in female *Disc1* svΔ2 animals compared with *Disc1* svΔ2 male animals suggests a structural predisposition to the psychiatric disease state in the latter. The findings comparing previous P84 results to the current study indicate that females harbor decreased neurite density at earlier points in development but then overcompensate beyond wild-type levels with increased neurite density and dispersion of neurite orientation. In contrast, *Disc1* svΔ2 males may be able to return neurite density to wild-type levels by adulthood but still retain significant deficits (complexity) in neurite orientation. This structural predisposition to the psychiatric disease state dovetails with the clinically observed increased prevalence of male psychopathy.

In addition to these statistical analyses comparing *Disc1* svΔ2 and control ROIs within male and female rats, additional ROI statistical analyses directly compared *Disc1* svΔ2 male rats to *Disc1* svΔ2 female rats. In concordance with both the above within-male and within-female ROI comparisons and with the existing literature examining general sex differences in the adult human brain, the direct comparison of *Disc1* svΔ2 male to *Disc1* svΔ2 female rats demonstrates that FA values are significantly lower in female rats than in males (Ritchie et al., 2018). Furthermore, ROI statistical analyses comparing *Disc1* svΔ2 male rats to *Disc1* svΔ2 female rats show that ODI values are significantly higher in female rats than in males, which aligns strongly with the finding in within-male and within-female ROI comparisons that *Disc1* svΔ2 male rats display significantly lower ODI than control males and *Disc1* svΔ2 female rats display significantly higher ODI values than control females.

With P120–P150 FA and NDI alterations tempered in *Disc1* svΔ2 male rats and NDI and ODI decreases reversed in *Disc1* svΔ2 female rats when compared with P84 subjects, the overall constellation of these findings provides additional experimental context for the wide variability in FA findings seen across human DTI studies of mental illness. Variability in study design, population characteristics, imaging modality parameters, and preprocessing steps all certainly contribute to the subsequent variability in study results seen in the literature; however, our study indicates that even with the application of highly homogenous cohorts, FA may be an insufficient tool to assess known neural microstructure alterations, especially in psychiatric illness. Given the rich literature of findings regarding reductions to FA in clinical studies of schizophrenia and other mental illnesses, it is clear that FA is a highly sensitive tool to observe gross white matter microstructure alteration, but at the same time is also relatively non-specific. Even if variability in patient study design were eliminated, advanced multicompartment DWI used in combination with FA analyses may be more capable of identifying salient results as demonstrated in our work herein. Multiple imaging sessions within longitudinal studies that can interrogate the dynamics of neural microstructure change over time can also be used to map the underlying pathology and trajectory of these neuropsychiatric disorders.

Previous behavioral analyses of other *Disc1* genetic mouse models have demonstrated heterogenous behavioral profiles, with some showing significantly higher locomotion in the open field and anxiety-related behavior in the elevated plus maze, with others finding no significant differences in open field locomotion, Y-maze alternation, or elevated plus-maze anxiety-related behavior (Clapcote et al., 2007; Hikida et al., 2007; Dachtler et al., 2016). One of these murine models used a missense mutation D453G in exon 5 of the mouse *Disc1* gene and observed evidence for disruption of GSK3β as well as anxiety-related behavior in *Disc1* mice of both sexes in the elevated-plus maze and open field. Estrogen-β-catenin interactions could potentially mediate this presentation of sexually dimorphic behavioral phenotypes that differ from the finding of deficits only in males in this current analysis. The finding of both sex-specific and endophenotypic category-spanning behavioral deficits in the *Disc1 svΔ2* rat model provides greater content validity and justification for using the *Disc1 svΔ2* model to interrogate the underlying biology of psychiatric illness. These findings of neural microstructure deficits in our *Disc1* svΔ2 model (global neural microstructure deficits at P84, global neural microstructure deficits and behavioral deficits at P120–P150) align with the currently understood time course of numerous psychiatric illnesses where structural alterations can be seen in the preclinical stages of the disease before the expression of the clinical phenotype.

In summary, this research illustrates the utility and value of NODDI and advanced DWI methods toward identifying and providing novel insights into neurodevelopmental trajectories as well as sex-specific changes in brain microstructure that cannot be differentiated by traditional diffusion tensor and morphometric analyses alone, while corroborating these changes with clinically relevant animal behavior. Sex-specific differences in neural microstructure and behavioral endophenotypes in the *Disc1 svΔ2* rat model clearly illustrate the greater extent of microstructural change present in male animals and behavioral deficits mirroring the greater male sex-specific incidence of psychiatric illness in patients with alterations to the *Disc1* locus. These efforts aid in understanding the degree of genetic susceptibility imparted by *Disc1* to both neural microstructure and behavioral deficits over the course of the neurodevelopmental timeline and the degree to which the environmental milieu can exacerbate or ameliorate the psychiatric disease state. The stark sex-specific differences and the age-related differences in endophenotypes observed in our analysis provides insight for the design of human neuroimaging studies of psychiatric illness and the import and value of NODDI over more traditional morphometric and DTI methods common employed. Considering biological sex as a separate experimental variable and selecting more specific age ranges creates cohort homogeneity that could more clearly delineate and disambiguate neural microstructural components and drivers of mental illness. Additionally, the combined application of more homogenous cohorts with dimensional traits such as NDI and ODI imaging parameters that more clearly correlate with clinical symptoms and behavioral endophenotypes can contribute to more complete neuropsychiatric subtype definitions and improve our capacity to understand disease risk and treatment options in psychiatric illness.

## Synthesis

Reviewing Editor: Francisca Bronfman, Universidad Andrés Bello Decisions are customarily a result of the Reviewing Editor and the peer reviewers coming together and discussing their recommendations until a consensus is reached. When revisions are invited, a fact-based synthesis statement explaining their decision and outlining what is needed to prepare a revision will be listed below. The following reviewer(s) agreed to reveal their identity: Elizabeth Hutchinson. Note: If this manuscript was transferred from JNeurosci and a decision was made to accept the manuscript without peer review, a brief statement to this effect will instead be what is listed below.

The manuscript has been sent to one of the previous reviewers of the JNeurosci version, several aspects of the methodology and subsequently, the results/discussions should be addressed to make this manuscript ready for publication:

Major points (both related to imaging methods):

1.

I notice your rebuttal response that TBSS “allows for whole brain voxel wise analysis (GM and WM) as was performed here. “ but this may not be true.

From Smith et al., 2006, page 1493, we learn that TBSS projects the <b>maximum</b> FA value perpendicular to the “tract” for each individual onto the mean FA skeleton and so is not really a “whole-brain” voxelwise technique in that it only evaluates the skeleton values, which are the highest FA values. Usually, the maximum FA value will be from a white matter voxel (also there is the FA<0.2 threshold to consider), so it is effectively a white-matter voxelwise approach (except maybe in the hippocampus or other high fa gm regions). Not a big deal, but a true voxelwise analysis of the DTI/NODDI maps (e.g. directly subjecting the merged FA/NDI/ODI/MD maps to randomize analysis with tfce) would be more complete by including potential gray matter findings, which may be considerable in the rodent brain as it has a higher relative GM volume.

If you have used a different implementation of TBSS that does not project the maximum FA, but rather some other value, please explain in the methods.

If you have used the standard TBSS methods, do you think it would have the following implications for your results/discussion points:

- Strain differences in maximum FA reported by TBSS are largely absent for males/females but strain differences in average FA are present and significant for ROI findings and in opposite directions. Consider revising lines 328-9 and 356-358 regarding improved FA specificity of ROIs.

- Better yet, consider performing a voxelwise analysis that considers all voxels (e.g. as described above). Your tensor-based registration should support such analysis and the results would be more appropriate for comparing to the ROI findings and better define the differences between ODI and FA.

Why is MD included in the ROI analysis, but not the TBSS/voxelwise analysis? At least for review it should be provided to show a dissimilar pattern of significant voxels from the NODDI metrics.

2.

Because the NODDI metrics are central to the focus of this paper, it is important to describe the specific changes that were made from the default clinical NODDI model/settings in the MDT software at a level that they could be performed in replication based on the methods.

The current wording is: Line 178 analytical pipelines are specifically designed for imaging data collected from fixed samples that account for the presence of fixative in the final model fitting

This is quite vague and suggests that the presence of fixative is in the model (that should not be true since you re-hydrated the sample and fixative will only indirectly affect this model via T2). In any case, the two main factors that have to be considered for this model in ex-vivo tissue are:

- Diffusivity assumptions are different due to the temperature at which the sample is imaged. The hard-coded/default values for clinical/in-vivo NODDI according to Zhang 2012, pg 1004 are d∥=1.7×10−3mm2s−1 and diso=3.0×10−3mm2s−1.

The recommendation for ex-vivo NODDI is to use your axial diffusivity in the corpus callosum for d∥ (around 0.6x10-3mm2/s) and the diffusivity of water at the temperature of your scanner bore for diso (around 2x10-3mm2/s).

Please confirm the values for these that were applied in your modeling.

- The model recommended for ex-vivo by the MIG (creators of NODDI) is ‘WatsonSHStickTortIsoVIsoDot_B0’, it is different from the default in-vivo/clinical because it includes a restricted compartment (that is more prominent in fixed tissue from the cross-linking). Did you use a model with this compartment? If not, why not and what model did you use?

** note: there are good discussions and suggestions on these points via the NODDI google groups page. They may be helpful to you. Or perhaps you have all of this information and simply omitted it from the text?

NODDI is quite sensitive to these two factors (for more details on this point see Guerrero et al., 2019; Jelescu and Budde, 2017; Hutchinson et al., 2017), so it is not a minor point and must be addressed. If a clinical/in-vivo model or parameters were used, they must be changed and the analysis re-performed. Please also provide a representative or average DTI and NODDI maps either as an additional paper figure (a good suggestion to accompany your introductory explanation of NODDI...) or minimally for the review process.

I have no major comments about the biological model or other content. The paper is well written and if the imaging methods can be confirmed/addressed, I think this work will be a meaningful contribution to the literature in the field.

Minor comments

Discussion:

Line 339 incomplete sentence

Lines 341-342 “deficient axonal pruning” seems a very specific mechanistic interpretation given how non-specific FA/MD can be, are there a few references you could include to support that this happens? Or additional hypotheses.

Lines 342-344 Given that this is white matter, do you mean axons instead of neurites? What do you mean by local circuits here?

## Author Response

Response to Reviewer Comments:

Mapping sex-specific neurodevelopmental alterations in neurite density

and morphology in a rat genetic model of psychiatric illness

We thank both the reviewers and Reviewing Editor for their careful consideration of our work and thoughtful

suggestions to improve our manuscript. In response to all comments, we have revised our manuscript to incorporate all recommended suggestions.

In the updated version of the text, these changes can be found in the ‘tracked changes’ version of our revision.

Point by point responses to the consensus recommendations follow below.

Convergent Review:

Major points (both related to imaging methods):

1. I notice your rebuttal response that TBSS “allows for whole brain voxel wise analysis (GM and WM) as was

performed here.” but this may not be true.

From Smith et al., 2006, page 1493, we learn that TBSS projects the maximum FA value perpendicular to the

"tract” for each individual onto the mean FA skeleton and so is not really a “whole-brain” voxelwise technique

in that it only evaluates the skeleton values, which are the highest FA values. Usually the maximum FA value

will be from a white matter voxel (also there is the FA<0.2 threshold to consider), so it is effectively a whitematter voxelwise approach (except maybe in the hippocampus or other high fa gm regions). Not a big deal, but

a true voxelwise analysis of the DTI/NODDI maps (e.g. directly subjecting the merged FA/NDI/ODI/MD maps to

randomize analysis with tfce) would be more complete by including potential gray matter findings, which may

be considerable in the rodent brain as it has a higher relative GM volume. If you have used a different implementation of TBSS that does not project the maximum FA, but rather some other value, please explain in the

methods.

The authors appreciate these very thoughtful comments and close review of the imaging methodologies of our

work. With regards to TBSS, we agree with the reviewer in their description of the technique and the authors

acknowledge the misleading language used in our manuscript. We did not use a different implementation of

TBSS and in light of these comments and the reviewer’s suggestion, we have now corrected all instances of

our incorrect nomenclature in the manuscript. Thank you.

If you have used the standard TBSS methods, do you think it would have the following implications for your

results/discussion points:

- Strain differences in maximum FA reported by TBSS are largely absent for males/females but strain differences in average FA are present and significant for ROI findings and in opposite directions. Consider revising

lines 328-9 and 356-358 regarding improved FA specificity of ROIs.

- Better yet, consider performing a voxelwise analysis that considers all voxels (e.g. as described above). Your

tensor-based registration should support such analysis and the results would be more appropriate for comparing to the ROI findings and better define the differences between ODI and FA.

The authors thank the reviewer again for these thoughtful and insightful comments and additional text is now

included in the Discussion (lines 368-370) regarding the improved FA specificity of ROIs. The authors also appreciate the suggestion of the reviewer to consider performing a new voxelwise analysis that considers all

voxels; as interesting as such a technical analysis would be, new information garnered from this analysis does

2

not substantive change the overall conclusions of our work, which is focused on a systems and clinical translational understanding of sex-specific behavioral and microstructural alterations in our small animal model. In our

estimation, the differences that we discovered, especially at the ROI level, have greater clinical salience for

systems and translational neuroscientists and while the approach as suggested by the author would likely better define and clarify potential subtle differences between voxelwise differences ODI and FA, we respectfully

suggest that the additive value of this new analysis is limited in the context of our current work. Thank you for

providing this considerate suggestion, which we anticipate to be a point of further exploration in other ongoing

projects in our laboratory.

Why is MD included in the ROI analysis, but not the TBSS/voxelwise analysis? At least for review it should be

provided to show a dissimilar pattern of significant voxels from the NODDI metrics.

MD analysis was included in the last revision as this was explicitly requested by a previous reviewer; the accompanying TBSS analysis of MD findings was not requested at that time. However, like the FA TBSS results,

there are no significant voxelwise differences present in the MD TBSS analysis.

2. Because the NODDI metrics are central to the focus of this paper, it is important to describe the specific

changes that were made from the default clinical NODDI model/settings in the MDT software at a level that

they could be performed in replication based on the methods.

The current wording is: Line 178 analytical pipelines are specifically designed for imaging data collected from

fixed samples that account for the presence of fixative in the final model fitting.

This is quite vague and suggests that the presence of fixative is in the model (that should not be true since you

re-hydrated the sample and fixative will only indirectly affect this model via T2). In any case, the two main factors that have to be considered for this model in ex-vivo tissue are:

- Diffusivity assumptions are different due to the temperature at which the sample is imaged. The hardcoded/default values for clinical/in-vivo NODDI according to Zhang 2012, pg 1004 are d∥ =1.7×10−3mm2s−1

and diso=3.0×10−3mm2s−1. The recommendation for ex-vivo NODDI is to use your axial diffusivity in the corpus callosum for d∥ (around 0.6x10-3mm2/s) and the diffusivity of water at the temperature of your scanner

bore for diso (around 2x10-3mm2/s).

Please confirm the values for these that were applied in your modeling.

These values were used in our modeling and for clarification, these additional methodological details have now

been added to the manuscript for clarity and reproducibility. Thank you.

- The model recommended for ex-vivo by the MIG (creators of NODDI) is ‘WatsonSHStickTortIsoVIsoDot_B0’,

it is different from the default in-vivo/clinical because it includes a restricted compartment (that is more prominent in fixed tissue from the cross-linking). Did you use a model with this compartment? If not, why not and

what model did you use?

The ‘WatsonSHStickTortIsoVIsoDot_B0 is the model we used as recommended by MIG.

** note: there are good discussions and suggestions on these points via the NODDI google groups page. They

may be helpful to you. Or perhaps you have all of this information and simply omitted it from the text?

NODDI is quite sensitive to these two factors (for more details on this point see Guerrero et al., 2019; Jelescu

and Budde, 2017; Hutchinson et al., 2017), so it is not a minor point and must be addressed. If a clinical/in-vivo

model or parameters were used, they must be changed and the analysis re-performed. Please also provide a

3

representative or average DTI and NODDI maps either as an additional paper figure (a good suggestion to accompany your introductory explanation of NODDI...) or minimally for the review process.

The authors appreciate this suggestion from the reviewer; yes, we are aware of the NODDI google groups

page and have used it extensively as a resource to guide our experimental work. All requested information is

now included in our manuscript at the excellent suggestion of this reviewer.

We also have numerous close collaborations with imaging groups that are interested in understanding and expanding the technical parameters of the NODDI model (Andy Alexander, PhD, UW-Madison, as cited by the

reviewer and their recent paper in PLoS One) and our collaborations with them have also informed the work

presented here. As authors, we have strived to frame this manuscript not as a narrowly tailored imaging study

but one placed more broadly as a systems neuroscience paper that aims to unite imaging with animal behavior

and partly explains why we did not include an average DTI or NODDI map in the manuscript proper (as might

be more befitting of a traditional imaging paper). We attached representative diffusion maps here below for the

reviewer’s consideration below (left to right: DWI, NDI, ODI). Thank you.

I have no major comments about the biological model or other content. The paper is well written and if the imaging methods can be confirmed/addressed, I think this work will be a meaningful contribution to the literature

in the field.

The authors thank the reviewer for sharing these comments, which have greatly clarified and improved our

manuscript. Thank you.

Minor comments

Discussion:

Line 339 incomplete sentence

The authors apologize for this oversight and have corrected this incomplete sentence.

Lines 341-342 “deficient axonal pruning” seems a very specific mechanistic interpretation given how nonspecific FA/MD can be, are there a few references you could include to support that this happens? Or additional hypotheses.

Additional citations/clarification is now provided. Thank you for bringing this to our attention.

4

Lines 342-344 Given that this is white matter, do you mean axons instead of neurites? What do you mean by

local circuits here?

We have clarified this sentence to state “axons” instead of neurites. The reference to local circuits is vague and

confusing and we have now removed this. Thank you.
